# Lipid and lipoprotein predictors of functional outcomes and long-term mortality after surgical sepsis

**DOI:** 10.1186/s13613-021-00865-x

**Published:** 2021-05-20

**Authors:** Faheem W. Guirgis, Christiaan Leeuwenburgh, Lyle Moldawer, Gabriela Ghita, Lauren Page Black, Morgan Henson, Elizabeth DeVos, David Holden, Phil Efron, Srinivasa T. Reddy, Frederick A. Moore

**Affiliations:** 1 Department of Emergency Medicine, University of Florida College of Medicine, Jacksonville 655 West 8th Street, Jacksonville, FL 32209 USA; 2 Department of Aging and Geriatric Research, University of Florida College of Medicine, Gainesville, FL USA; 3 Department of Surgery, University of Florida College of Medicine, Gainesville, FL USA; 4 Department of Biostatistics, University of Florida College of Public Health and Health Professions, Gainesville, USA; 5 Department of Medicine, David Geffen School of Medicine, University of California Los Angeles, Los Angeles, CA 90095 USA

**Keywords:** Sepsis, Lipid metabolism, Critical illness

## Abstract

**Rationale:**

Sepsis is a life-threatening, dysregulated response to infection. Lipid biomarkers including cholesterol are dynamically regulated during sepsis and predict short-term outcomes. In this study, we investigated the predictive ability of lipid biomarkers for physical function and long-term mortality after sepsis.

**Methods:**

Prospective cohort study of sepsis patients admitted to a surgical intensive-care unit (ICU) within 24 h of sepsis bundle initiation. Samples were obtained at enrollment for lipid biomarkers. Multivariate regression models determined independent risk factors predictive of poor performance status (Zubrod score of 3/4/5) or survival at 1-year follow-up.

**Measurements and main results:**

The study included 104 patients with surgical sepsis. Enrollment total cholesterol and high-density lipoprotein (HDL-C) levels were lower, and myeloperoxidase (MPO) levels were higher for patients with poor performance status at 1 year. A similar trend was seen in comparisons based on 1-year mortality, with HDL-C and ApoA-I levels being lower and MPO levels being higher in non-survivors. However, multivariable logistic regression only identified baseline Zubrod and initial SOFA score as significant independent predictors of poor performance status at 1 year. Multivariable Cox regression modeling for 1-year survival identified high Charlson comorbidity score, low ApoA-I levels, and longer vasopressor duration as predictors of mortality over 1-year post-sepsis.

**Conclusions:**

In this surgical sepsis study, lipoproteins were not found to predict poor performance status at 1 year. ApoA-I levels, Charlson comorbidity scores, and duration of vasopressor use predicted 1 year survival. These data implicate cholesterol and lipoproteins as contributors to the underlying pathobiology of sepsis.

**Supplementary Information:**

The online version contains supplementary material available at 10.1186/s13613-021-00865-x.

## Introduction

Sepsis is a dysregulated response to infection that leads to organ dysfunction and potentially death [1]. Sepsis mortality is substantial at 20% worldwide, though this figure is improved from prior decades [2]. However, focusing on initial survival alone is not adequate for understanding patient-oriented outcomes after sepsis. Our group and others have demonstrated that the long-term outcomes of early sepsis survivors are frequently poor [3, 4]. Sepsis survivors frequently experience persistent and long-term organ dysfunction as well as recurrent sepsis [4, 5]. In addition, a subset of patients (up to 40%) develop a syndrome of chronic critical illness (CCI), defined as ICU stay ≥ 14 days with continued organ dysfunction, and clinically result in lean muscle wasting, cachexia, and a 1-year mortality approaching 50% [3, 6, 7]. This group of patients requires high levels of post-discharge care, with most of them experiencing limitations in performing activities of daily living, and frequently limited to a bed or chair for most of the day [3].

The term persistent inflammation immunosuppression and catabolism syndrome (PICS) was coined to describe the presumed underlying pathobiology of CCI that occurred in over one-third surgical ICU patients treated for sepsis [7]. It is characterized by persistent inflammation (elevation of IL-6 and IL-8 levels), immunosuppression [lymphopenia, elevated soluble programmed death ligand-1 (sPDL-1), and nosocomial infections], and catabolism [8]. Compared to medical ICU sepsis patients, the PICS-CCI phenotype may be more common in surgical ICU sepsis patients for several reasons. First, because of surgeon selection bias surgery, patients have lower likelihood of having severe comorbidities and thus have early sepsis care terminated for perceived futility. Second, surgical patients frequently undergo source control interventions that can dramatically improve organ dysfunction and short-term survival. Third, surgical patients may require repeat source controls interventions and have surgical wounds that prolong inflammation and dysregulated immunity. These observations have been described and elucidated in our prior publications, from a large prospective observational study of surgical sepsis [9–11].

Endogenous lipids and lipoproteins are highly metabolically active during sepsis and critical illness [12, 13]. High-density lipoprotein (HDL) and low-density lipoprotein (LDL) have both been shown to have protective roles in sepsis [14, 15]. HDL is a major source of cholesterol for endogenous corticosteroid production in the adrenal glands [16, 17], and prevents tissue damage in sepsis, a function mediated by several of HDLs proteins including paraoxonase-1 (PON-1) and apolipoprotein A-I (Apo A-I) [13]. Toxins from both Gram-negative bacteria (endotoxin) and Gram-positive bacteria (lipoteichoic acid) are neutralized by HDL via Apo A-I [13, 18–21] and LDL has also been shown to bind and clear endotoxin via the liver [22–24]. However, we have shown that HDL becomes pro-inflammatory or dysfunctional (Dys-HDL) when subjected to inflammation in sepsis, and that Dys-HDL correlates with and predicts organ failure severity in sepsis [25]. We have also demonstrated disparate outcomes associated with cholesterol levels and function, with the most severe organ failure being associated with the lowest early cholesterol levels [26, 27]. Based on these and other studies, we hypothesize that an interplay between inflammation, organ failure, and lipid metabolism exists that is predictive of poor performance status and long-term mortality after sepsis [25, 28]. The objectives of the proposed of study were to evaluate the predictive ability of early lipid indicators for poor performance status and 1-year mortality after surgical sepsis.

## Methods

### Design

This study was a subgroup analysis of a 5-year prospective, longitudinal cohort study of patients with surgical sepsis. Patients were included in the subgroup analysis if they had data on lipid and oxidative biomarkers sent as part of the prospective study. Additional file 1: Tables S1 and Additional file 2: Table S2 compare the subgroup cohort to the overall cohort. Surgical sepsis was defined as patients admitted to a surgical or trauma ICU who were treated for sepsis with our institutional evidence-based sepsis bundle. Patient enrollment was approved by the University of Florida Institutional Review Board and registered with *clinicaltrials.gov* (NCT02276417).

### Patient selection and enrollment

UF Health Gainesville patients admitted to the surgical ICU and entered into the standard-of-care sepsis protocol and meeting Sepsis-2 criteria were considered potential study candidates for the P50 sepsis study. Sepsis-2 criteria were used as the study began in 2015 prior to the development of Sepsis-3. Interim analysis of this cohort in 2017 demonstrated that only 7% of study patients that were classified as sepsis by Sepsis-2 would have been reclassified as infection by Sepsis-3 (because of lack attributable organ dysfunction) [29]. When various equivalent strata of Sepsis-2 and Sepsis-3 cohorts were compared, no significant difference in immune biomarkers, SOFA scores, inpatient clinical outcomes, discharge disposition, mortality, and long-term Zubrod performance status were found. A study research nurse assessed patients for study criteria and consented and enrolled appropriate candidates 24 h/day, 7 days a week [30]. A detailed description of the study design with specific inclusion and exclusion criteria as well as the clinical and laboratory standard operating procedure (SOPs) utilized has been published [29]. Exclusion criteria eliminated patients whose baseline immunosuppression, end-stage comorbidities, or severe functional disabilities would be a primary determinant of their long-term outcomes and thus confound outcome assessment.

### Enrollment blood sampling and testing

While hospitalized, blood was drawn 12 h after enrollment on the first day of the study. Measures of lipid and lipoprotein levels and function included the following: 1) cholesterol levels, 2) HDL function and oxidation status (cell-free assay for Dys-HDL), and 3) HDL anti-oxidant activity (PON-1, ApoA-I). Myeloperoxidase (MPO), a pro-oxidant peroxidase enzyme released by neutrophils for bacterial killing, was also measured as it is known to contribute to oxidation of HDL and ApoA-I [31]. PON-1 is an HDL-associated esterase that protects lipoproteins, most likely by hydrolyzing lipid peroxides including oxidized cholesteryl esters and phospholipids [32].

Serum total cholesterol, HDL-C, and triglyceride levels were directly measured from serum samples. LDL-C was calculated using the Friedewald formula from total cholesterol, HDL-C, and triglyceride [33]. Dys-HDL and PON-1 activity were measured and reported as in prior studies [27]. Briefly, the cell-free assay for Dys-HDL requires HDL isolation from blood samples using dextran sulfate precipitation and LDL prepared from a normal donor. After incubating experimental (patient HDL plus control LDL) and control subject samples (control LDL only) with dichlorofluorescein, the ability of sample HDL to protect LDL from oxidation was quantitated by the decline in fluorescence and expressed as a ratio of the fluorescence released, the HDL inflammatory index (HII) [28]. PON-1 activity was quantified using paraoxon as the substrate and measuring the increase in absorbance at 405 nm due to the formation of 4-p-nitrophenol over a period of 12 min (at 20-s intervals). Paraoxon was purchased from Sigma-Aldrich (St. Louis, MO) and was further purified using chloroform extraction. One unit of PON-1 activity was defined as the formation of 1 nmole of 4-p-nitrophenol per minute per milliliter of sample used. Enzyme activity was measured with 40-fold-diluted plasma (final concentration) in a reaction mixture containing 4 mM paraoxon working solution, 500 mM glycine/10 mM CaCl2 buffer at pH 10.5, and 155 mM NaCl/3 mM NaN3 buffer at pH 8.2 [34]. Quantikine™ ELISA kits (R&D Systems, Inc, Minneapolis, MN) were used to measure plasma Apo A-I. Plasma MPO levels were measured using the MPO ELISA kit from Mercodia (Sweden).

### Performance status assessments

Performance status was assessed using the ECOG/WHO/Zubrod Scale. Zubrod is calculated based on a 5-point scale that measures the performance status of a patients ambulatory abilities: 0, asymptomatic (fully active); 1, symptomatic but completely ambulatory (restricted in physically strenuous activity); 2, symptomatic, less than 50% in bed during the day (ambulatory and capable of all self-care but unable to perform any work activities); 3, symptomatic, greater than 50% in bed, but not bedbound (capable of only limited self-care); 4, bedbound (completely disabled, incapable of any self-care); and 5, death [3, 8]. Pre-sepsis baseline Zubrod scores were established via patient or surrogate interviews at 3 month follow-up visits and independently adjudicated by three members of the research team. Zubrod estimates were completed for participants with missing follow-up data based on qualitative notes taken during monthly phone calls or during clinic visits.

### Data collection

All data were entered by trained research nurses and coordinators with investigator oversight. Study data were prospectively collected and included demographics, place of residence, source of infection, and Charlson comorbidities and score. Clinical variables including enrollment vital signs, timing of antibiotics, volume of intravenous fluids administered in the first six and 24 h, vasopressor use and duration in hours, mechanical ventilation use, central venous pressures (CVP), and urine output in the first six hours. Home medications including statin use were recorded. Admission disposition, hospital length of stay (LOS), and ICU LOS were documented. On the 4^th^ day of admission, repeat clinical assessments were performed including repeat vital signs, hemodynamic and ventilator requirements, and SOFA score. Chart reviews after enrollment were performed to confirm source of infection and sepsis diagnosis, culture results, ICU and hospital LOS, and discharge disposition. Mortality information was acquired by monthly follow-up phone calls or from the social security death index.

### Clinical outcomes and adjudication

The two primary outcomes of interest were (1) poor performance status outcomes at 1 year (defined as Zubrod score of 3/4/5) and (2) 1-year survival. Based on our previous studies, there were three short-term study endpoints (clinical trajectories) included: (1) incidence of early death (within 2 weeks of hospital admission), (2) CCI (total ICU stay > 14 days or total ICU ≤ 14 days but discharged to long-term acute care, another hospital, or hospice), or (3) rapid recovery (all others) [35]. Patients were further classified as having a good disposition if they were discharged to home or a rehabilitative facility, and poor disposition if they were discharged to LTAC, skilled nursing facility, another hospital, hospice, or died. The senior author (FAM) and his team of surgical intensivist co-investigators performed adjudications via weekly group adjudication meetings [36]. Data were only included for patients who had values for lipid biomarkers and functional outcomes measured included as part of the prospective study.

### Sample size and data analysis

The study was powered to detect a correlation between HII and SOFA score. We calculated that a sample size of 160 patients, accounting for 15% early mortality (resulting in at least 136 living), would have 84% power to detect a Pearson correlation coefficient of 0.25 between change in HII and change in SOFA score over the first 48–72 h. These correlations are not presented in this manuscript as they were previously published [25].

Data are presented as frequency and percentages or medians with interquartile ranges for categorical and continuous variables, respectively. Zubrod plots were created by plotting Zubrod score at enrollment, 3, 6, and 12 months by quartiles of lipid levels, functional, and MPO levels. The first quartile contained patients with the lowest biomarker levels, while the fourth quartile had patients with the highest biomarker levels. We used Fisher’s exact test for comparison of categorical variables and the Kruskal–Wallis test for comparison of continuous variables. The log-rank test was used to compare Kaplan–Meier product limit estimates of survival between groups. Following univariate analysis, multivariate stepwise logistic regression models were used to determine independent risk factors predictive of (1) poor performance status (Zubrod 3/4/5) at 1 year and (2) 1-year survival. ApoA-I values were large, and therefore, log-transformed values were used in statistical modeling to generate interpretable confidence intervals. Multivariate stepwise cox regression models were used to determine factors associated with 1-year survival time. For both logistic regression and Cox regression models, we first examined the association between various parameters of interest and the outcomes of poor physical function and mortality at 1-year follow-up, respectively. Parameters found statistically significant on univariate analysis were considered for inclusion in the multivariate models. We list the parameters considered for inclusion in each model below each respective section of Table 5. Stepwise selection was used to determine the final multivariable models with a *p* value of 0.1 used for entry and retention into the model. Otherwise, significance tests were two-sided, with *p* value ≤ 0.05 considered statistically significant. Statistical analyses were performed with SAS version 9.4 (Cary, NC; U.S.A.).

## Results

The study included 104 patients with surgical sepsis. Patient demographics and reason for hospital admission for this cohort are depicted in Table 1. The same comparisons to 363 patients enrolled to the larger P50 grant study are depicted in Additional file 1: Table S1. There was no demonstrable selection bias for this sub-study compared to the overall cohort. The median age of the 104 study patients was 63 years (IQR 51, 72), there were more males than females (55%), and patients were predominantly White. The most common comorbidities were hypertension, followed by diabetes mellitus, and coronary artery disease. The majority of patients were admitted for an active infection (60%), followed by planned surgery (20%) as the second most common reason for hospital admission. A large proportion of patients were transferred from other institutions (41%).

**Table 1:** Demographics, comorbidities, and reason for admission

Variable	Entire Cohort (*n* = 104)	1-year survivors* (*n* = 74)	1-year non-survivors* (*n* = 24)	*P* value
Age in years, median (25th, 75th)	63 (51, 72)	60 (44, 70)	69 (56, 77)	0.026
Male, *n* (%)	57 (55)	38 (51)	15 (63)	0.36
Race,* n* (%)				0.19
Caucasian (White)	96 (92)	67 (91)	24 (100)	
African American	8 (8)	7 (10)	0 (0)	
Statin use,* n* (%)	33 (32)	22 (30)	8 (33)	0.76
Charlson Comorbidity Index, median (25th, 75th)	3 (1, 4)	2 (0, 4)	4 (3, 5)	0.002
Comorbidities,* n* (%)				
Solid cancer	13 (13)	8 (11)	4 (17)	0.48
Hematologic cancer	0 (0)	0 (0)	0 (0)	N/A
Dementia	4 (4)	4 (5.4)	0 (0)	0.57
Heart failure	9 (9)	7 (10)	2 (8)	1
Liver disease	2 (2)	2 (3)	0 (0)	1
Chronic lung disease	15 (14)	11 (15)	4 (17)	1
Chronic renal disease	14 (13)	9 (12)	4 (17)	0.73
Prior stroke	11 (11)	7 (10)	3 (13)	0.70
Coronary disease	27 (26)	17 (23)	9 (38)	0.19
Diabetes	37 (36)	27 (37)	9 (38)	1
Substance abuse	4 (6)	4 (5)	0 (0)	0.57
Hypertension	70 (67)	49 (66)	16 (67)	1
Atrial fibrillation	15 (14)	9 (12)	4 (17)	0.73
Morbid obesity	21 (20)	16 (22)	4 (17)	0.77
Peripheral artery disease	7 (7)	4 (5)	3 (13)	0.36
Reason for hospital admission,* n* (%)				0.60
Planned surgery	20 (19)	15 (20)	4 (17)	
Trauma	11 (11)	10 (14)	1 (4)	
Active infection	62 (60)	42 (57)	16 (67)	
Non-infectious/chronic problems	11 (11)	7 (10)	3 (13)	
Inter-facility transfer,* n* (%)	43 (41)	28 (38)	12 (50)	0.34

*indicate that 6 patients were lost to follow up and did not have data on 1-year survival

Table 2 displays data on source of infection, disease severity, and surgical interventions. The most common infection type was surgical site infection, followed by intra-abdominal, and urinary tract. Just over half of patients were culture positive. Most patients had sepsis present at the time of admission, while 41% had hospital-acquired infections. Median Apache II and SOFA scores were high, reflecting the severity of illness as all patients were admitted to the ICU. Just over half of patients had an emergency surgery within 24 h of hospital presentation, and two-thirds had a source control procedure at some point in their hospital stay.

**Table 2:** Sepsis diagnosis, illness severity, and surgical interventions

Variable	Entire Cohort (*n* = 104)	1-year survivors* (*n* = 74)	1-year non-survivors* (*n* = 24)	*P* value
Primary sepsis Diagnosis,* n* (%)				0.29
Intra-abdominal	24 (23)	14 (19)	10 (42)	
Central line- associated blood- stream infection	2 (2)	1 (1)	1 (4)	
Necrotizing soft- tissue infection	9 (9)	6 (8)	1 (4)	
Pneumonia	14 (14)	10 (14)	4 (17)	
Surgical site Infection	25 (24)	20 (27)	4 (17)	
Urosepsis	18 (17)	14 (19)	2 (8)	
Other	12 (12)	9 (12)	2 (8)	
Culture status,* n* (%)				0.22
Negative	35 (34)	23 (31.1)	11 (46)	
Positive	69 (66)	51 (69.0)	13 (54)	
Gram-positive	16 (15)	11 (14.9)	4 (17)	
Gram-negative	30 (29)	22 (29.7)	5 (21)	
Fungal	3 (3)	3 (4.1)	0 (0)	
Polymicrobial	20 (19)	15 (20.3)	4 (17)	
Vasopressors given,* n* (%)	36 (35)	19 (25.7)	15 (63)	0.003
Duration of vasopressor use (hours), median (25th, 75th)	0 (0, 14)	0 (0, 1)	24 (0, 60)	0.0001
Sepsis present on admission,* n* (%)	61 (59)	41 (55)	14 (58)	1
Hospital-acquired sepsis,* n* (%)	43 (41)	33 (45)	10 (42)	1
Initial lactate, median (25th, 75th)	1.7 (1.2, 3)	1.6 (1.1, 2.4)	2.75 (1.6, 4.5)	0.012
Repeat lactate, median (25th, 75th)	1.4 (0.9, 2.2)	1.3 (0.9, 1.8)	2.2 (1.2, 3.5)	0.007
Max lactate within 24 h of onset, median (25th, 75th)	2.4 (1.6, 4.1)	2.1 (1.5, 3.2)	3.85 (2.5, 9.2)	0.0004
APACHE II Score (24 h), median (25th, 75th)	19 (13, 25)	17.5 (11, 24)	24.5 (19.5, 28.5)	0.0008
SOFA score (24 h), median (25th, 75th)	6.5 (5, 10)	6 (4, 9)	11 (8, 13)	< .0001
Sepsis severity,* n* (%)				0.018
Sepsis	26 (25)	22 (30)	3 (13)	
Severe sepsis	47 (45)	35 (47)	8 (33)	
Septic shock	31 (30)	17 (23)	13 (54)	
Emergency surgery within 24 h,* n* (%)	55 (53)	41 (55)	11 (46)	0.484
Source control procedure,* n* (%)	70 (67.3)	50 (68)	17 (71)	1
Source control procedure type,* n* (%)				0.52
Invasive	51 (49)	34 (46)	14 (58)	
Non-invasive	19 (18.3)	16 (22)	3 (13)	

*indicate that 6 patients were lost to follow up and did not have data on 1-year survival

Table 3 displays data on inpatient care and patient outcomes. The median hospital length of stay (LOS) was nearly 2 weeks and most patients were on mechanical ventilation at some point during the hospitalization. A surprising small number of patients died early (7%). The majority of patients had rapid recovery (60%), however, of note just over one-third progressed into CCI. Sixty percent of study patients were discharged with a good disposition (home or rehab facility), and 40% percent were discharged with a poor disposition (LTAC, skilled nursing facility, another hospital, hospice, or death). A substantial proportion of patients (37%) had poor performance status (Zubrod 3/4/5) 1 year after sepsis. Mortality at one year was 23%.

**Table 3:** Inpatient care, clinical trajectories, and outcomes

Variable	Entire Cohort (*n* = 104)	1-year survivors* (*n* = 74)	1-year non-survivors* (*n* = 24)	*P* value
Hospital LOS, median (25th, 75th)	15 (8, 27)	15 (9, 30)	18 (6, 26)	0.69
ICU LOS, median (25th, 75th)	7 (3, 15.5)	6 (3, 14)	15 (4.5, 20)	0.11
Need for mechanical ventilation,* n* (%)	64 (62)	42 (57)	21 (88)	0.007
Ventilator free days (30 days), median (25th, 75th)	28 (22, 30)	29 (26, 30)	2.5 (0, 25.5)	< .0001
Acute kidney injury,* n* (%)	69 (66)	49 (66)	17 (71)	0.804
Clinical trajectory,* n* (%)				< .0001
Early death (< 14 days)	7 (7)	0 (0)	7 (29)	
Chronic critical illness	35 (34)	19 (26)	15 (63)	
Rapid recovery	62 (60)	55 (74)	2 (8)	
Discharge disposition,* n* (%)				< .0001
“Good” disposition	62 (60)	53 (72)	5 (21)	< .0001
Home	17 (27)	14 (26)	1 (20)	
Home healthcare services	32 (52)	29 (55)	3 (60)	
Rehab	13 (21)	10 (19)	1 (20)	
“Poor” disposition	42 (40)	21 (28)	19 (79)	< .0001
Long term acute care facility	13 (31)	9 (43)	3 (16)	
Skilled nursing facility	10 (23.81)	9 (42.9)	0 (0)	
Another hospital	3 (7)	3 (14)	0 (0)	
Hospice	1 (2)	0 (0)	1 (5)	
Death	15 (36)	0 (0)	15 (79)	
30-day mortality,* n* (%)	16 (15)	0 (0)	16 (67)	< .0001
180-day mortality,* n* (%)	21 (20)	0 (0)	21 (87.5)	< .0001
Zubrod 3/4/5 at 1 year,* n* (%)	34 (37)	10 (14%)	24 (100%)	NA
Zubrod 4/5 at 1 year,* n* (%)	27 (26)	3 (4%)	24 (100%)	NA
1-year mortality,* n* (%)	24 (23)	0 (0%)	24 (100%)	NA

*indicate that 6 patients were lost to follow up and did not have data on 1-year survival

**Fig. 1:**
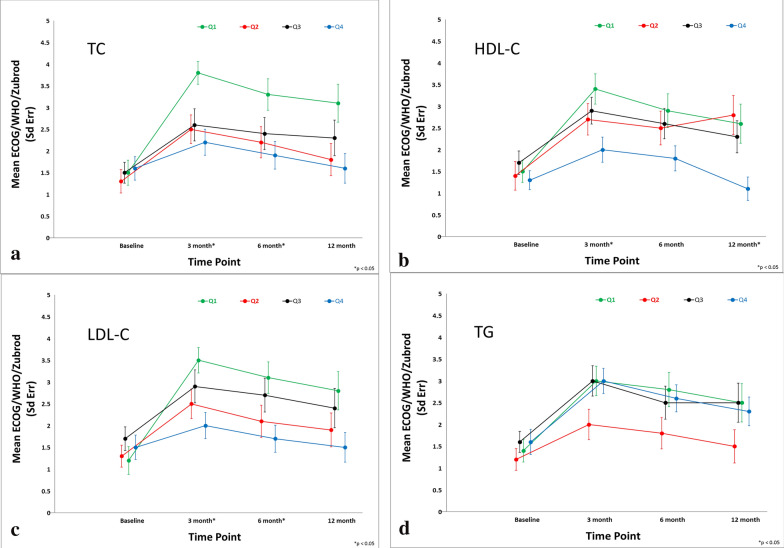

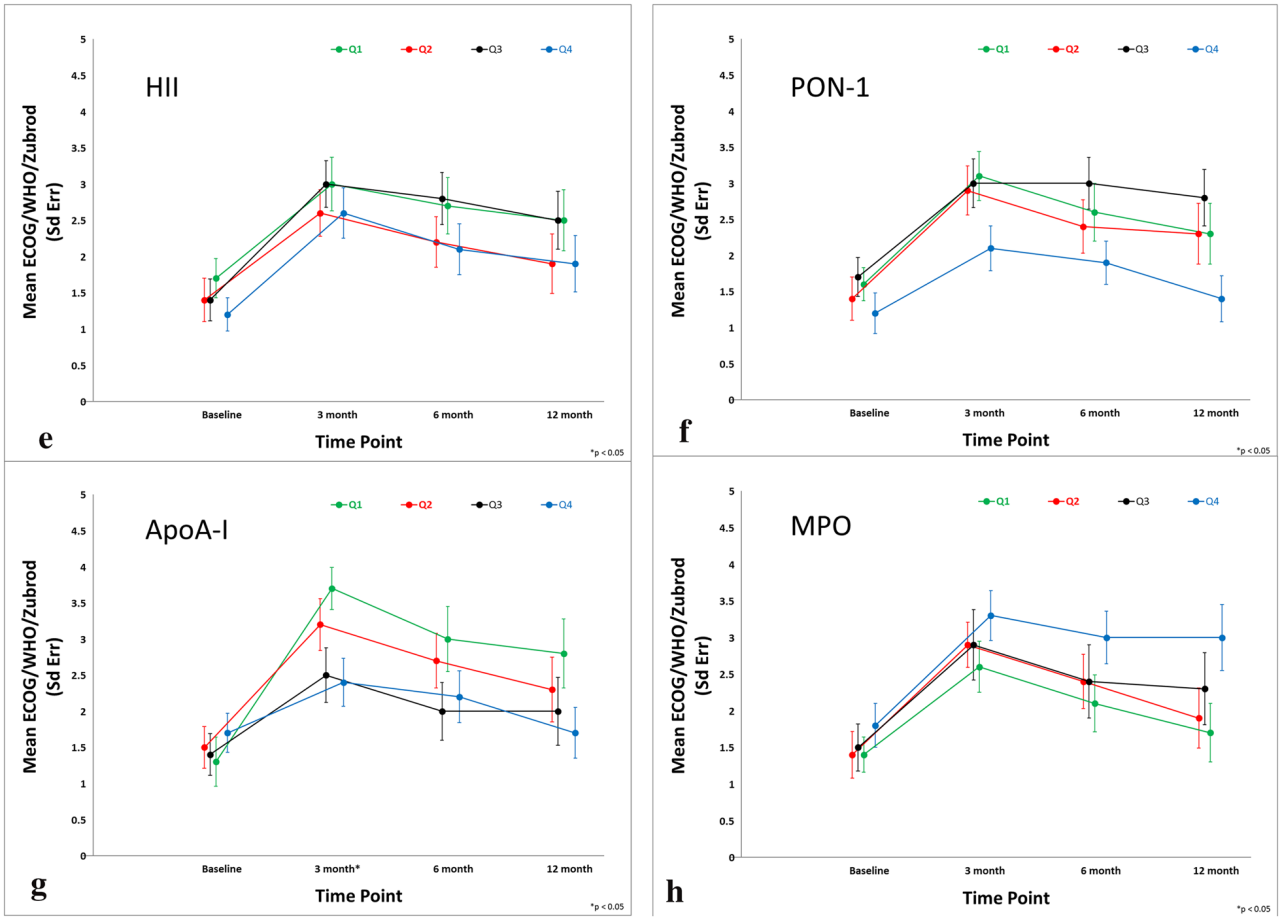
Quartiles of ECOG/WHO/Zubrod score plotted at baseline, 3, 6, and 12 months for: **a** TC (total cholesterol), **b** high-density lipoprotein-cholesterol (HDL-C), **c** low-density lipoprotein-cholesterol (LDL-C), **d** triglycerides (TG), **e** HDL inflammatory index (HII), **f** paraoxonase-1 (PON-1), **g** apolipoprotein A-I (ApoA-I), and **h** myeloperoxidase (MPO). Differences at each time point are represented by colored lines for lipid biomarker quartiles with standard error bars, with asterisks indicating significant differences per timepoint

Lipid levels, function, and oxidative biomarkers are presented in Table 4. Enrollment total cholesterol and high-density lipoprotein (HDL-C) levels were lower and myeloperoxidase (MPO) levels were higher for patients with poor performance status at 1 year. Plots of Zubrod scores at enrollment, 3, 6, and 12 months by lipid levels, function, and MPO level quartiles are displayed in Fig. 1. From Fig. 1, patients with the lowest quartile total cholesterol levels had significantly higher Zubrod scores at 3 and 6 months, while patients with higher HDL-C levels had lower Zubrod scores at 3 and 12 months. Similar trends were seen with LDL-C levels, in which lower quartiles were associated with higher Zubrod scores at 3 and 6 months. Lower ApoA-I levels were also associated with higher Zubrod scores at 3 months. A similar trend was seen in comparisons based on 1-year mortality, with HDL-C and ApoA-I levels being lower and MPO levels being higher in non-survivors.

**Table 4:** Lipids levels, function, and oxidative markers

Lipid or oxidative marker at enrollment	Zubrod 0–2 (*n* = 59)	Zubrod 3–5 (*n* = 34)	*P* value
Total cholesterol, median (25th, 75th)	90 (67, 117)	70.5 (53, 107)	0.040
HDL-C, median (25th, 75th)	19 (11, 26)	14 (6, 21)	0.028
LDL-C, median (25th, 75th)	43 (27, 65)	38 (21, 58)	0.28
Triglycerides, median (25th, 75th)	118 (82, 144)	120.5 (75, 149)	0.92
PON-1, median (25th, 75th)	42.22 (22.7, 90.8)	33.38 (21.88, 65.47)	0.20
HII, median (25th, 75th)	1.52 (1.03, 2.96)	1.52 (0.98, 2.59)	0.65
ApoA-I, median (25th, 75th)	1,000,000 (746,140, 1,400,000)	880,377 (654,664, 1,200,000)	0.15
MPO, median (25th, 75th)	184.92 (128.6, 318.8)	297.77 (178.2, 489.3)	0.025

Lipid or oxidative marker at enrollment	1-year survivors* (*n* = 74)	1-year non-survivors* (*n* = 24)	*P* value
Total cholesterol, median (25th, 75th)	84 (64, 114)	70 (46, 105)	0.051
HDL-C, median (25th, 75th)	18 (9, 26)	9 (6, 20)	0.031
LDL-C, median (25th, 75th)	41.5 (27.5, 62)	36 (21, 58)	0.39
Triglycerides, median (25th, 75th)	112 (82, 149)	120.5 (73, 134)	0.82
PON-1, median (25th, 75th)	49.36 (25.2, 78.5)	29.9 (21.4, 64.9)	0.09
HII, median (25th, 75th)	1.5 (1.03, 2.8)	1.52 (0.97, 2.6)	0.71
ApoA-I, median (25th, 75th)	1,000,000 (746,140, 1,400,000)	817,243 (654,397, 978,674)	0.024
MPO, median (25th, 75th)	190.09 (138.7, 332.6)	301.6 (183.4, 508.7)	0.040

*indicate that 6 patients were lost to follow up and did not have data on 1-year survival

**Fig. 2:**
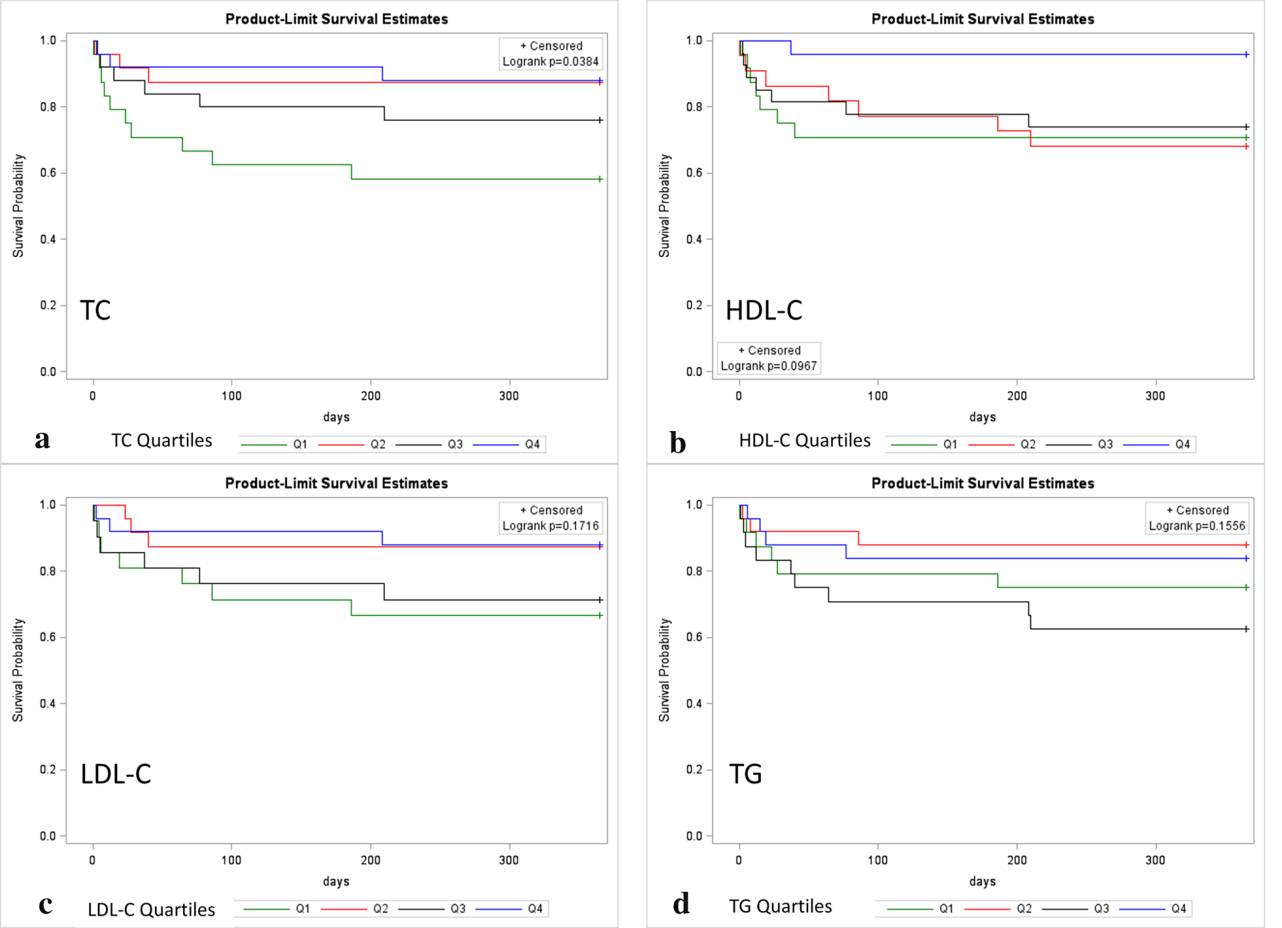

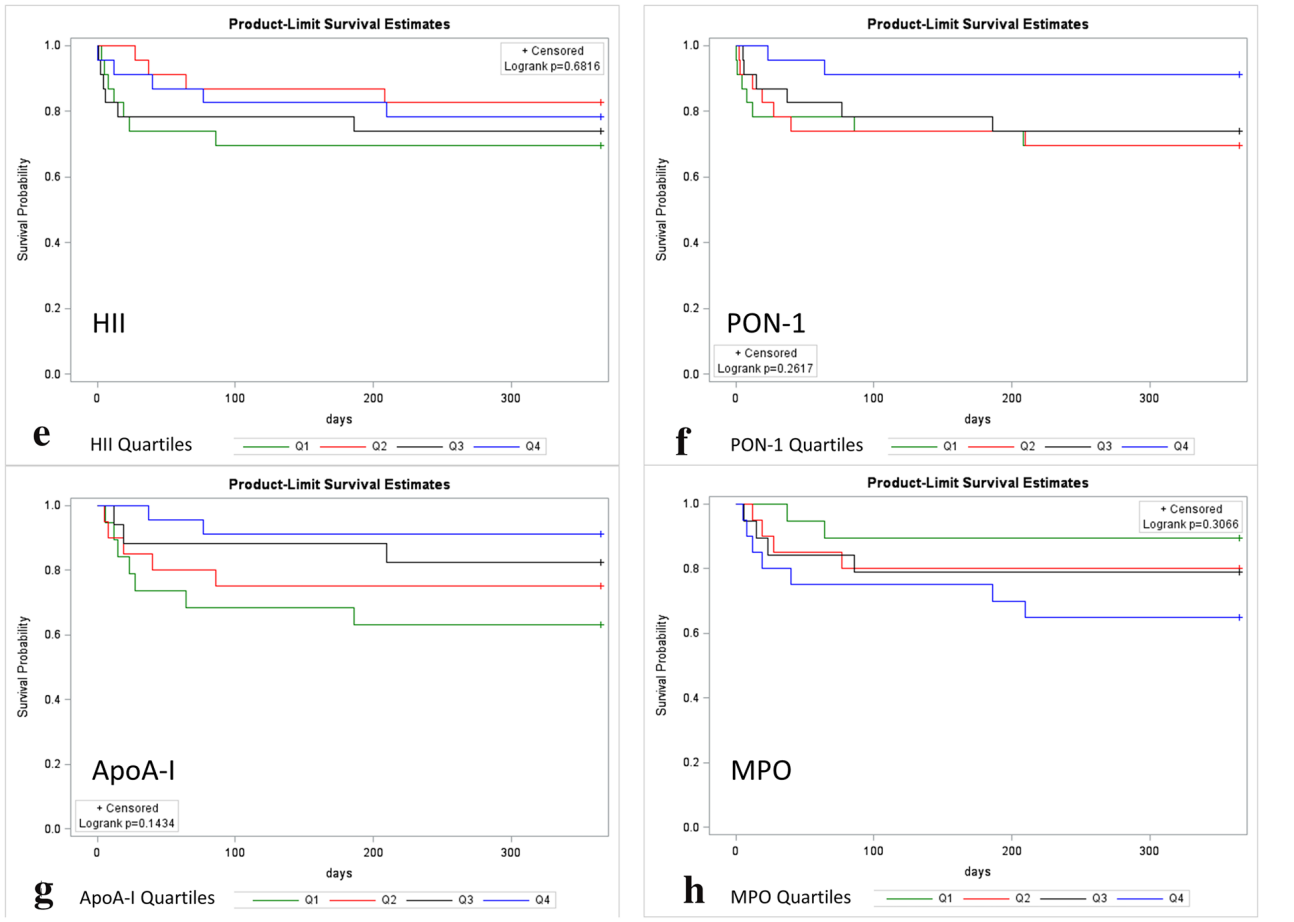
: Kaplan–Meier survival curves over 1 year after sepsis are presented by quartiles for **a** TC (total cholesterol), **b** high-density lipoprotein-cholesterol (HDL-C), **c** low-density lipoprotein-cholesterol (LDL-C), **d** triglycerides (TG), **e** HDL inflammatory index (HII), **f** paraoxonase-1 (PON-1), **g** apolipoprotein A-I (ApoA-I), and **h** myeloperoxidase (MPO)

After adjusting for other covariates including disease severity, multivariable logistic regression identified baseline Zubrod score and initial SOFA score as significant independent predictors of poor performance status at 1 year. Survival curves based on quartiles of lipid levels, function, and oxidative biomarkers are presented in Fig. 2. Multivariable Cox regression modeling for 1-year survival identified high Charlson comorbidity score, low ApoA-I levels, and longer vasopressor duration use as predictors of mortality over 1 year. Regression model results are presented in Table 5.

**Table 5:** Prediction models for Zubrod 3/4/5 and 1-year survival

Predictor	OR	OR lower 95% CL	OR Upper 95% CL	*P* value
Multivariate logistic regression models predicting Zubrod 3/4/5 at 1-year follow-up^a^				
Baseline Zubrod	2.147	1.244	3.705	0.0061
SOFA (24 h)	1.225	1.023	1.467	0.0271

Predictor	HR	HR lower 95% CL	HR Upper 95% CL	*P* value
Multivariate Cox regression modeling survival over 1 year				
Charlson Comorbidity Score	1.315	0.998	1.732	0.03
ApoA-I (log)	0.315	0.094	1.056	0.059
Vasopressor duration	1.04	1.017	1.063	0.0003

^a^AUC: 0.7763 (95% CI: 0.6445, 0.9081)

Parameters considered for inclusion: Baseline Zubrod score, T0 total cholesterol, T0 HDL-C, T0 MPO level, Charlson Comorbidity score, Vasopressor duration, vasopressor given, volume of fluids in first 24 h, SOFA (24 h)

Parameters considered for inclusion: T0 HDL-C, Charlson Comorbidity score, ApoA-I (log), T0 MPO, T0 lactate, mechanical ventilation use, Vasopressor duration, vasopressors given, volume of fluids in first 24 h, SOFA (24 h)

## Discussion

In this study of lipid biomarkers in patients with surgical sepsis, only Apo A-I was a significant independent predictor of 1-year survival. None of our lipid markers were found to be significant independent predictors of poor performance status at 1 year. After adjustment for other factors, only baseline Zubrod score and SOFA score predicted poor performance status. In the multivariable Cox regression model, higher Charlson score, longer duration of vasopressor use, and lower ApoA-I levels predicted 1-year mortality. Both greater chronic comorbidities and septic shock are known to portend poor long-term outcomes both in terms of organ function and mortality [4, 35, 37].

To our knowledge, this is the first study to evaluate the relationship between lipid biomarkers and physical performance status and long-term morbid outcomes in surgical sepsis patients. Functional outcomes and long-term mortality are highly relevant outcomes for modern sepsis research as in-hospital survival has improved due to advances in sepsis management. Recently, early recognition and evidence-based care have led to improved short-term outcomes [38–40]. However, a large proportion of patients are left with considerable residual morbidity, and much of the burden of disease is transferred to the post-hospital setting where mortality and physical function data are not reported [3, 4]. Our group has recently demonstrated the breadth and severity of poor functional outcomes after surgical sepsis across the spectrum of patients, and has shown the discordance between seemingly low short-term mortality rates and the prevalence of poor performance status and high mortality at 1 year [3, 35, 41] This may be attributed to PICS as the underlying pathobiology of the CCI phenotype, where inflammation, immunosuppression, and catabolism contribute to these outcomes. In surgical sepsis, repeated exposures to inflammation-propagating procedures contribute to the maladaptive cycle of persistent inflammation involving reduced host protective immunity, continued organ injury, which include loss of muscle mass and function, change in bone marrow function, and failure of metabolic adaptation [9]. In this study, we had the ability to perform long-term follow-up assessments on our patients, which provided valuable data on quality of life and mortality after discharge. Of the patients who survived hospital discharge, roughly half of the patients either had poor physical function or were dead 1 year after sepsis. Emergency surgery contributes to and perpetuates this vicious cycle, which likely contributes to the very high incidence of CCI and poor physical function reported in this study.

Lipid biomarkers have long been shown to be highly associated and predictive of outcomes in sepsis [42, 43]. Low LDL-C, total cholesterol, and HDL-C levels have been associated with increased sepsis risk and poor outcomes [28, 42–45]. High-density lipoprotein-cholesterol (HDL-C) is protective against sepsis by several mechanisms including bacterial toxin binding [13, 18], suppression of inflammatory cell migration, prevention of inflammatory cytokine release [46, 47], inhibition of vascular and intercellular adhesion molecules expression [31, 48], and stimulation of endogenous corticosteroid release [16, 17]. ApoA-I has been shown to be a potent anti-oxidant and anti-inflammatory, a mechanism that may provide an explanation for the association of low levels with poor sepsis outcomes. In a recent study of rats, an ApoA-I mimetic peptide (4F) was administered to rats with induced sepsis via cecal ligation and puncture. Rats treated with 4F were found to have suppression of sepsis-induced changes in serum cholesterol, LDL, HDL, and ApoA-I levels, reduced inflammation, and protection of cardiac and renal function [49]. The use of HDL and ApoA-I mimetics has been proposed as potential therapeutic options for improving sepsis outcomes [14, 49, 50]. Others have focused on LDL-C and its ability to bind and clear endotoxin via hepatocytes in models of Gram-negative sepsis [22–24]. Specific to this idea is the concept of PCSK9 inhibition, and the resultant maintenance of LDL-C receptors on the surface of hepatocytes allowing for increased endotoxin clearance from circulation and elimination via the biliary tract. This implies the potential use of PCSK9 inhibitors to improve outcomes from sepsis, though this has not been tested.

In this study, we report results of the functional and anti-oxidant status of HDL. The HII provides an index of HDL’s oxidation status, while PON-1 activity and ApoA-I levels provide data on HDL’s function as an anti-oxidant. MPO activity, which is known to play a role in oxidizing HDL, was also reported. Though HII and PON-1 activity were not significantly different for patients with poor functional outcomes and long-term mortality, MPO and ApoA-I levels were significantly different in univariate comparisons, while only ApoA-I was predictive in multivariable analyses. MPO is elevated in infection and has been shown to contribute to the generation of dysfunctional HDL with impaired cholesterol efflux mediated by HDL [51]. The overall body of literature in this arena supports our findings that ApoA-I may be central to the dysregulated metabolism of sepsis.

In our review, we were unable to find other studies reporting on the predictive ability of lipids for long-term physical function in sepsis survivors. There are a few studies, however, that report on lipid prediction of long-term outcomes after sepsis. Wei and colleagues recently showed the predictive ability of low HDL-C levels and ApoA-I levels in predicting long-term mortality in patients with infective endocarditis [52]. Genga and colleagues also recently reported that patients with low HDL had greater frequency of worsened acute kidney injury, and long-term decreases in glomerular filtration rate (eGFR) up to 2 years after sepsis [53]. We have previously shown that low LDL-C levels, after adjusting for other factors, lead to increased sepsis risk over the subsequent years in a study of 30,000 community dwelling adults in the Reasons for Geographic and Racial Differences in Stroke (REGARDS) study [26]. Future studies should explore the mechanisms of lipid metabolism and dysregulation, the presence of bioactive lipid metabolites over time, and the genomic changes in lipid regulatory genes to deepen our understanding of lipid dysregulation in sepsis. In addition, studies should continue to focus on physical performance status as a meaningful indicator of sepsis recovery as well as long-term mortality.

There were two main limitations to this study. First, this was a small cohort of patients with surgical sepsis. Because of the small sample size, we may have been underpowered to detect additional differences and lipid predictors that would have been detected in a larger sample of patients. Second, this was a study of surgical sepsis patients that were treated for predominantly surgical infections. As such, these outcomes may not be necessarily generalized to a broader sepsis population and findings should be validated in a large more diverse cohort.

## Conclusions

In this study of patients with surgical sepsis, ApoA-I was the only lipid or lipoprotein biomarker found to be predictive of 1-year mortality after adjusting for other variables including disease severity. None of our lipid or lipoprotein biomarkers were found to be predictive of poor performance status at 1 year. Large studies in a more diverse cohort of community and hospital-acquired sepsis patients should be undertaken to validate these findings. Mechanistic studies should also be performed to better understand mechanisms of lipid dysregulation in sepsis.

## Supplementary Information

**Additional file 1: Table S1.** Demographics of lipid study cohort vs. entire P50 cohort. APACHE, acute physiology and chronic health evaluation.

**Additional file 2: Table S2.** Outcomes of Lipid study cohort vs. entire P50 cohort. LOS, length of stay; ICU, intensive-care unit.

## Data Availability

Supporting data from this study can be obtained by emailing the corresponding author Dr. Faheem W. Guirgis, MD.
