# Supplementary material for: Lipid and lipoprotein predictors of functional outcomes and long-term mortality after surgical sepsis
Source: Ann Intensive Care. 2021 May 20;11:82. doi: 10.1186/s13613-021-00865-x (PMC8136376; doi:10.1186/s13613-021-00865-x)
Supplement: Supplementary file 1 — Additional file 1: Table S1. Demographics of lipid study cohort vs. entire P50 cohort. APACHE, acute physiology and chronic health evaluation. [file 13613_2021_865_MOESM1_ESM.docx]

| **Supplemental Table 1. Demographics of Lipid study cohort vs. entire P50 cohort** | | |
| --- | --- | --- |
| **Variable** | **Overall (n=104)** | **P50 (n=363)** |
| Male, n (%) | 57 (55) | 196 (54) |
| Age in years, median (25th, 75th) | 63 (51, 72) | 62 (50, 71) |
| Race, n (%) |  |  |
| Caucasian | 96 (92) | 324 (90) |
| African American | 8 (8) | 35 (10) |
| American Indian | 0 (0) | 1 (0.3) |
| Asian | 0 (0) | 1 (0.3) |
| Other | 0 (0) | 1 (0.3) |
| Unknown | 0 (0) | 1 (0.3) |
| Charlson Comorbidity Index, median (25th, 75th) | 3 (1, 4) | 3 (1, 5) |
| APACHE II, median (25th, 75th) | 19 (13, 25) | 17 (11, 23) |
| Inter-facility hospital transfer, n (%) | 43 (41) | 152 (42) |
| Comorbidities, n (%) |  |  |
| Solid cancer | 13 (13) | 57 (16) |
| Hematologic cancer | 0 (0) | 1 (0.3) |
| Dementia | 4 (4) | 8 (2) |
| Heart failure | 9 (9) | 47 (13) |
| Liver disease | 2 (2) | 10 (3) |
| Chronic lung disease | 15 (14) | 70 (19) |
| Chronic renal disease | 14 (13) | 50 (14) |
| Prior Stroke | 11 (11) | 26 (7) |
| Coronary disease | 27 (26) | 89 (25) |
| Diabetes | 37 (36) | 124 (34) |
| Substance abuse | 4 (6) | 33 (9) |
| Hypertension | 70 (67) | 226 (62) |
| Atrial fibrillation | 15 (14) | 42 (12) |
| Morbid obesity | 21 (20) | 65 (18) |
| Peripheral artery disease | 7 (7) | 44 (12) |
| Sepsis severity, n (%) |  |  |
| Sepsis | 26 (25) | 110 (30) |
| Severe sepsis | 47 (45) | 156 (43) |
| Septic shock | 31 (30) | 97 (27) |
| Primary Sepsis Diagnosis, n (%) |  |  |
| Intra-abdominal | 24 (23) | 105 (29) |
| Central line-associated blood  stream infection | 2 (2) | 5 (1.4) |
| Necrotizing soft tissue  infection | 9 (9) | 42 (12) |
| Pneumonia | 14 (14) | 60 (17) |
| Surgical site infection | 25 (24) | 84 (23) |
| Urosepsis | 18 (17) | 42 (12) |
| Other | 12 (12) | 25 (7) |
