# Supplementary material for: Lipid and lipoprotein predictors of functional outcomes and long-term mortality after surgical sepsis
Source: Ann Intensive Care. 2021 May 20;11:82. doi: 10.1186/s13613-021-00865-x (PMC8136376; doi:10.1186/s13613-021-00865-x)
Supplement: Supplementary file 2 — Additional file 2: Table S2. Outcomes of Lipid study cohort vs. entire P50 cohort. LOS, length of stay; ICU, intensive-care unit. [file 13613_2021_865_MOESM2_ESM.docx]

| **Supplemental Table 2.** **Outcomes of Lipid study cohort vs. entire P50 cohort** | | |
| --- | --- | --- |
| **Variable** | **Overall (n=104)** | **P50 (n=363)** |
| In-hospital mortality, n (%) | 15 (14) | 28 (8) |
| ICU LOS, median (25th, 75th) | 7 (3, 15.5) | 7 (3, 16) |
| Hospital LOS, median (25th, 75th) | 15 (8, 27) | 15 (8, 26) |
| Discharge disposition, n (%) |  |  |
| “Good” disposition | 62 (60) | 208 (57) |
| Home | 17 (16) | 72 (20) |
| Home healthcare services | 32 (31) | 110 (30) |
| Rehab | 13 (13) | 26 (7) |
| “Poor” disposition | 42 (40) | 157 (43) |
| Long Term Acute Care facility | 13 (13) | 54 (15) |
| Skilled Nursing facility | 10 (10) | 53 (15) |
| Another Hospital | 3 (3) | 13 (4) |
| Hospice | 1 (1) | 9 (3) |
| Death | 15 (14) | 28 (8) |
| 30-day mortality, n (%) | 16 (15) | 34 (10) |
| 12-month mortality, n (%) | 24 (23) | 78 (24) |
| Zubrod at 12 months, median (25th, 75th) | 2 (1, 5) | 1.5 (1, 5) |
